# HIV-1 Nef promotes migration and chemokine synthesis of human basophils and mast cells through the interaction with CXCR4

**DOI:** 10.1186/s12948-016-0052-1

**Published:** 2016-11-01

**Authors:** Francesca Wanda Rossi, Nella Prevete, Felice Rivellese, Antonio Lobasso, Filomena Napolitano, Francescopaolo Granata, Carmine Selleri, Amato de Paulis

**Affiliations:** 1 Department of Translational Medical Sciences and Center for Basic and Clinical Immunology Research (CISI), University of Naples Federico II, Via S. Pansini 5, 80131 Naples, Italy; 2 Centre for Experimental Medicine and Rheumatology, William Harvey Research Institute, Barts and The London School of Medicine and Dentistry, Queen Mary University of London, London, UK; 3 Hematology Branch, Department of Medicine, University of Salerno, Salerno, Italy

**Keywords:** Mast Cells, Basophils, Nef, CXCR4, CXCL12/SDF-1α

## Abstract

**Background:**

The Nef protein can be detected in plasma of HIV-1-infected patients and plays a role in the pathogenesis of HIV-1. Nef produced during the early stages of infection is fundamental in creating the ideal environment for viral replication, e.g. by reducing the ability of infected cells to induce an immune response.

**Aim:**

Based on previous experience showing that both Tat and gp41 of HIV-1 are potent chemotactic factors for basophils and mast cells, and gp120 is a powerful stimulus for the release of histamine and cytokines (IL-4 and IL-13) from basophils, in this study we aimed to verify if the HIV Nef protein can exert some effects on basophils and mast cells purified from healthy volunteers through the interaction with the CXCL12 receptor, CXCR4.

**Methods:**

Basophils purified from peripheral blood cells of 30 healthy volunteers and mast cells obtained from lung tissue of ten healthy volunteers were tested by flow cytometric analysis, chemotaxis and chemokine production by ELISA assays.

**Results:**

Nef is a potent chemoattractant for basophils and lung mast cells obtained from healthy, HIV-1 and HIV-2 seronegative individuals. Incubation of basophils and mast cells with Nef induces the release of chemokines (CXCL8/IL-8 and CCL3/MIP-1α). The chemotactic activity of Nef on basophils and mast cells is mediated by the interaction with CXCR4 receptors, being blocked by preincubation of FcεRI^+^ cells with an anti-CXCR4 Ab. Stimulation with Nef or CXCL12/SDF-1α, a CXCR4 ligand, desensitizes basophils to a subsequent challenge with an autologous or heterologous stimulus.

**Conclusions:**

These results indicate that Nef, a HIV-1-encoded α-chemokine homolog protein, plays a direct role in basophils and mast cell recruitment and activation at sites of HIV-1 replication, by promoting directional migration of human FcεRI^+^ cells and the release of chemokines from these cells. Together with our previous results, these data suggest that FcεRI^+^ cells contribute to the dysregulation of the immune system in HIV-1 infection.

## Background

The human immunodeficiency viruses HIV-1 and HIV-2 destroy CD4^+^ lymphocytes, thus leading to AIDS [1]. Entry of HIV-1 into immune cells is mediated by the viral envelope glycoproteins (gp120 and gp41) [2] through their interaction with the CD4 glycoprotein, the primary receptor [3], the CC chemokine receptor 5 (CCR5) and the CXC chemokine receptor 4 (CXCR4), obligate coreceptors for virus entry [2].

Viral replication and host defence escape are regulated by HIV-1 proteins. The accessory protein Nef, is a crucial determinant of viral pathogenesis and disease progression to full-blown AIDS by optimizing the cellular environment for viral replication [4]. The key role of Nef is to control the expression levels of various cell surface molecules that play important roles in immunity and virus life cycle [5]. For example, Nef upregulates the surface expression of Tumor Necrosis Factor (TNF) and immature major histocompatibility complex class II (MHC-II). In contrast, Nef downregulates the surface expression of several other proteins including CD4, MHC-I, CD3, CD8, CD28, CXCR4, CCR5, CCR3, CD1, CD80/CD86, CTLA-4, mature (antigenic peptide-loaded) MHC-II [6]. Nef-mediated downregulation of MHC-I molecules, benefits the virus by interfering with the recognition and destruction of infected cells by cytotoxic T-cells [6]. Besides its well-studied effects on intracellular signaling, Nef also acts through its secretion in exosomes nanovesicles. Nef enhances exosome secretion and entry into uninfected CD4^+^ T cells, thus leading to apoptotic death [7]. Nef is also responsible for the inhibition of T cell migration in vitro [8]. In addition, Nef affects the innate immune system by impairing phagocytosis, and augmenting the release of pro-inflammatory and chemotactic factors from macrophages [9]. Altogether, Nef activities support viral replication and survival while at the same time favor viral dissemination [10]. Many of these activities of extra-cellular Nef might be mediated indirectly or directly by the interaction with the chemokine receptor CXCR4 [2, 11, 12].

Basophils and mast cells are the only cells synthesizing histamine and expressing high affinity receptors for IgE (FcεRI) [13]. Immunologic activation of human basophils leads to the release of proinflammatory mediators and the synthesis of a restricted profile of cytokines (IL-4 and IL-13) and chemokines (CXCL8/IL-8 and CCL3/MIP-1α) [14, 15], while human mast cells express a wide spectrum of cytokines and chemokines [16, 17]. Besides being the effector cells of IgE-mediated responses, basophils and mast cells are implicated in many physiological and pathological processes, such as the response to infections [18, 19], inflammatory and autoimmune diseases [20, 21] and cancer [22, 23].

We have investigated the role of basophils and mast cells in the context of HIV infection, suggesting that FcεRI^+^ cells may be a source of Th2 cytokines, thus contributing to the dysregulation of the immune system in HIV-1. Tat protein is a potent chemoattractant for human basophils and mast cells by interacting with the α-chemokine receptor CCR3 [24]. HIV-1 envelope gp41 peptide promotes migration of basophils and mast cells through interaction with formyl peptide receptors (FPRs) [25] and HIV-1 gp120 is a potent stimulus for IL-4 and IL-13 release from basophils [26, 27]. More recently, it has been reported that human mast cells can act as an inducible reservoir of persistent HIV infection [28] and that both mucosal mast cells and blood circulating basophils capture HIV-1 mediating viral trans-infection through the expression of multiple attachment factors (HAFs) [29, 30]. These findings indicate that human basophils and mast cells can contribute to the spread and persistence of HIV infection.

The results of our study further highlight the multiple interactions between HIV products and FcεRI^+^ cells and confirm the relevance of these cells in the promotion of HIV-1 infection.

## Methods

### Purification of peripheral blood basophils

Basophils were purified from peripheral blood cells of 30 healthy, HIV-1 and HIV-2 seronegative, volunteers, aged 20–39 years (mean, 33.6 ± 4.9 years). Buffy coat cell packs from healthy volunteers, provided by the Hematology Unit of the University of Salerno, were reconstituted in PBS containing 0.5 g/L HSA and 3.42 g/L sodium citrate, and loaded onto a countercurrent elutriator (model J2-21; Beckman, Fullerton, CA). Several fractions were collected, and fractions containing large numbers of basophils (>20 × 10^6^) and of good purity (>15%) were enriched by discontinuous Percoll gradients [16]. Basophils were further purified to near homogeneity (>98%) by depleting B cells, monocytes, NK cells, dendritic cells, erythrocytes, platelets, neutrophils, eosinophils, and T cells with a cocktail of hapten-conjugated CD3, CD7, CD14, CD15, CD16, CD36, CD45RA, and anti-HLA-DR Abs and MACS MicroBeads coupled to an anti-hapten mAb. The magnetically labeled cells were depleted by retaining them on a MACS column in the magnetic field of the MidiMACS (Miltenyi Biotec, Bergisch Gladbach, Germany). Yields ranged from 3 to 10 × 10^6^ basophils, with purity usually >98%, as assessed by basophil staining with Alcian Blue and counting in a Spiers-Levy eosinophil counter.

### Isolation and purification of human lung mast cells (HLMC)

Lung tissue was obtained from ten patients undergoing thoracotomy and lung resection, after obtaining their informed consent according to the guidelines of the institutional review board. Macroscopically normal parenchyma was dissected free from pleura, bronchi, and blood vessels and minced into a single-cell suspension as previously described [31]. Yields ranged between 3 × 10^6^ and 18 × 10^6^ mast cells, with purity between 1 and 8%. Lung mast cells were purified by countercurrent elutriation (J2/21; Beckman) and then by discontinuous Percoll density gradient as previously described [31]. Mast cells were further purified to near homogeneity by positive selection and incubation with anti-FcεRI (IgG1) followed by the exposure to magnetic beads coated with MACS goat anti-mouse IgG. Labeled cells were enriched by positive selection columns (MACS system; Miltenyi Biotec). The final preparations contained >95% viable cells, as assessed by the trypan blue exclusion method, and purity was >98% mast cells.

### Flow cytometric analysis of surface molecules

Flow cytometric analysis of cell surface molecules was performed as previously described [32]. Briefly, after saturation of non specific binding sites with total rabbit IgG, cells were incubated for 20 min at +4 °C with specific or isotype control antibodies. For indirect staining this step was followed by a second incubation for 20 min at +4 °C with an appropriate anti-isotype-conjugated antibody. Finally, cells were washed and analyzed with a FACSCalibur Cytofluorometer using Cell Quest software (Becton & Dickinson, San Fernando, CA). A total of 10^4^ events for each sample were acquired in all cytofluorimetric analyses.

### Chemotaxis assay

Basophil and mast cell chemotaxis was performed using a modified Boyden chamber technique as previously described [33]. Briefly, 25 µl of a Ca^2+^-containing buffer or various concentrations of the chemoattractants in the same buffer were placed in triplicate in the lower compartment of a 48-well microchemotaxis chamber (Neuroprobe, Cabin John, MD). The lower compartments were covered with polycarbonate membranes with 5-µm pores (basophils) or with a two-filter sandwich constituted by 5-µm (lower) and 8-µm (upper) pore size polycarbonate membranes (mast cells) (Nucleopore, Pleasanton, CA). Fifty microliters of the cell suspensions (5 × 10^4^/well) resuspended in a Ca^2+^-containing buffer was pipetted into the upper compartments. The chemotactic chamber was then incubated for 1 h (basophils) or 3 h (mast cells) at 37 °C in a humidified incubator with 5% CO_2_ (automatic CO_2_ incubator, model 160 IR, ICN/Flow Laboratories). At the end of basophil incubation, the membrane was removed, washed with PBS on the upper side, fixed, and stained with May-Grunwald/Giemsa. When mast cells were used, the upper polycarbonate filter was discarded, while the lower nitrate cellulose filter was fixed in methanol, stained with Alcian Blue, and then mounted on a microscope slide with Cytoseal (Stephen Scientific, Springfield, NJ). Basophil and mast cell chemotaxis was quantitated microscopically by counting the number of cells attached to the surface of the 5-µm cellulose nitrate filter. In each experiment 10 fields/triplicate filter were measured at ×40 magnification. The results were compared with buffer controls.

### *IL*-*4, IL*-*13, CXCL8/IL*-*8, CCL3/MIP*-*1*α *ELISA*

IL-4, IL-13, CXCL8/IL-8, CCL3/MIP-1α release in the culture supernatants of basophils and HLMC cells were measured in duplicate determinations with a commercially available ELISA kit (R&D System, Minneapolis, MN) [32].

### Statistical analysis

The results are expressed as the mean ± SEM. Statistical significance was analyzed by one-way ANOVA and, when the F value was significant, by Duncan’s multiple range test [34]. Differences were considered significant at *p* < 0.05.

## Results

### CXCR4 expression on human basophils and mast cells

**Fig. 1:**
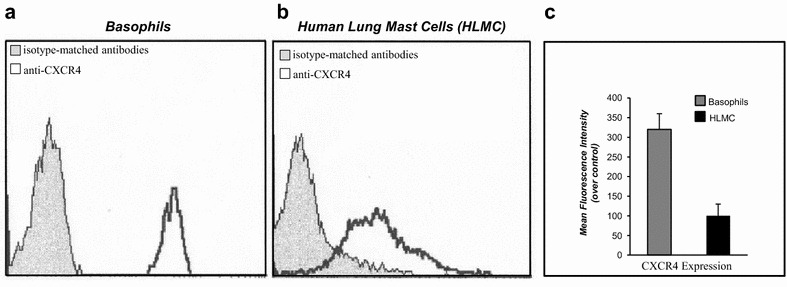
CXCR4 expression on human basophils and mast cells. **a** Cytofluorimetric analysis of CXCR4 expression by human basophils purified from normal donors, HIV-1 and HIV-2 seronegative. Basophils were incubated (25 °C, 45 min) with monoclonal anti-CXCR4 PerCP-labelled (5 µg/ml) and anti-IgE FITC-labelled (*white histogram*) or isotype-matched antibodies (*grey histogram*). **b** Cytofluorimetric analysis of CXCR4 expression by HLMC cells purified from normal donors, HIV-1 and HIV-2 seronegative. Mast cells were incubated (25 °C, 45 min) with monoclonal anti-CXCR4 PerCP-labelled (5 µg/ml) and anti-IgE FITC-labelled (*white histogram*) or isotype-matched antibodies (*grey histogram*). **c** Mean fluorescence intensity of CXCR4 expression in basophils (*grey column*) and HLMC cells (*black column*) over basal

Extracellular Nef exerts several functions on immune cells via CXCR4 receptors [11, 12, 35]. We have therefore investigated at protein level, by flow cytometry, the expression of CXCR4 on human basophils and mast cells. Figure. 1 shows that the vast majority of basophils (~80%) (Fig. 1a) and HLMC (~65%) (Fig. 1b) expressed on their surface the chemokine receptor CXCR4. Figure 1c shows the mean fluorescence intensity of CXCR4 expression in basophils (grey column) and HLMC cells (black column) over basal.

### Effect of HIV-1 r-Nef protein on human basophil *and mast cell* chemotaxis

**Fig. 2:**
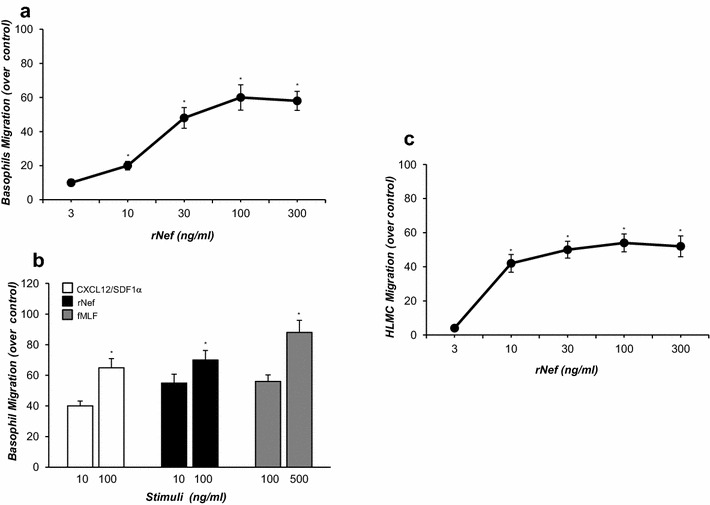
Effect of r-Nef on chemotaxis of human basophils and mast cells. **a** Basophils were allowed to migrate toward r-Nef protein (3-300 ng/ml) for 1 h at 37 °C in the humidified incubator with 5% CO_2_. Values are the mean ± SEM obtained from six independent experiments with different human basophil preparations. *p < 0.05 as compared to control. **b** Basophils were allowed to migrate toward the indicated concentrations of CXCL12/SDF-1α (*white histogram*), r-Nef (*black histogram*), and fMLF (*grey histogram*) for 1 h at 37 °C in a humidified incubator with 5% CO_2_. Values are the mean ± SEM obtained from four experiments. *p < 0.05 as compared to control. **c** HLMC cells were allowed to migrate toward r-Nef protein (3-300 ng/ml) for 3 h at 37 °C in the humidified incubator with 5% CO_2_. Values are the mean ± SEM obtained from six different experiments. *p < 0.05 as compared to control

Having found that FcεRI^+^ cells expressed the chemochine receptor CXCR4, we then assessed whether Nef was able to induce the chemotaxis of these cells. Figure 2a shows that r-Nef (3–300 ng/ml) (Abcam, Milton, Cambridge, UK) caused a concentration-dependent increase in chemotaxis of purified basophils. In a parallel series of experiments we compared the chemotactic activity of r-Nef with that of CXCL12/SDF-1α (R&D System (Minneapolis, MN) and of the formylated tripeptide N-formyl-methionyl-leucyl-phenylalanine (fMLF) (ICN Biomedicals) potent chemoattractants of human basophils through their interaction with the chemokine receptor CXCR4 and FPR1, respectively [19, 33]. Figure 2b shows that CXCL12/SDF-1α (10 and 100 ng/ml) and fMLF (100 and 500 ng/ml) induced strong chemotaxis of human basophils. In the same experiments r-Nef (10 and 100 ng/ml) promoted comparable migratory effects on basophils.

Since a remarkable proportion of HLMC cells (65%) expressed CXCR4 receptor (Fig. 1b) and CXCR4 receptor on human mast cells was functionally active being involved in the chemotactic response to CXCL12/SDF-1α, we tested the chemotactic response to r-Nef of HLMC cells. Figure. 2c shows that r-Nef (3–300 ng/ml) induced a concentration-dependent increase in HLMC cells chemotaxis.

Checkerboard analysis was performed to discriminate between chemotaxis and nondirectional migration (chemokinesis) of basophils or mast cells. Cell migratory responses to specific stimuli were largely due to chemotaxis and not to chemokinesis (data not shown).

### Nef-induced migration of basophils and mast cells through CXCR4

**Fig. 3:**
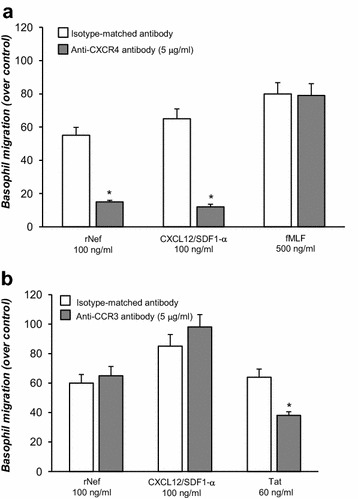
Effect of preincubation with anti-CXCR4 and anti-CCR3 antibody on r-Nef-dependent human basophil chemotaxis **a** Basophils were incubated with (*grey histogram*) or without (*white histogram*) anti-CXCR4 antibody (5 µg/ml) for 1 h, then loaded into the chemotaxis chamber and allowed to migrate toward the indicated concentrations of r-Nef, CXCL12/SDF-1α and fMLF for 1 h at 37 °C in a humidified incubator with 5% CO_2_. Values are the mean ± SEM of three distinct experiments. *p < 0.05 as compared to control. **b** Basophils were incubated with (*grey histogram*) or without (*white histogram*) anti-CCR3 antibody (5 µg/ml) for 1 h, then loaded into the chemotaxis chamber and allowed to migrate toward the indicated concentrations of r-Nef, CXCL12/SDF-1α and Tat protein for 1 h at 37 °C in a humidified incubator with 5% CO_2_. Values are the mean ± SEM of three distinct experiments. *p < 0.05 as compared to control

To establish whether the expression of CXCR4 on basophils was responsible for the chemoattractant effect of Nef, basophils were preincubated with an anti-CXCR4 antibody (5 μg/ml) and then assessed for their ability to migrate in response to Nef. Figure 3a shows that preincubation of basophils with an anti-CXCR4 antibody (R&D System, Minneapolis, MN) (5 μg/ml) inhibited the chemoattractant effect of Nef. Similarly, preincubation of basophils with an anti-CXCR4 antibody completely suppressed the chemotactic activity of CXCL12/SDF-1α (100 ng/ml) on these cells. In contrast, the chemotactic effect of fMLF (500 ng/ml), which activates a specific seven-transmembrane receptor independent of the CXCR4 receptor [33, 36], was not affected by the anti-CXCR4 antibody.

We have previously demonstrated that Tat protein was an HIV-1-encoded α-chemokine homologous that promotes basophil migration through the interaction with the chemokine receptor CCR3 [24]. Figure 3b demonstrate that preincubation of basophils with anti-CCR3 antibody (R&D System, Minneapolis, MN) (5 μg/ml) inhibited the chemoattractant effect of Tat (60 ng/ml). In contrast, the chemotactic effects of both CXCL12/SDF-1α (100 ng/ml) and r-Nef (100 ng/ml) were not affected by anti-CCR3 antibody. In similar experiments, preincubation of mast cells with a monoclonal antibody against CXCR4 completely blocked the chemoattractant effect of Nef protein (data not shown).

### Nef-induced heterologous desensitization *of CXCR4*

**Fig. 4:**
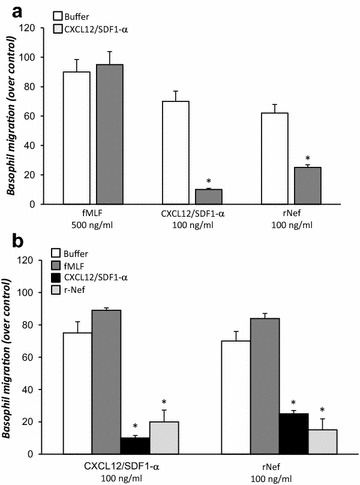
Nef-induced heterologous desensitization of CXCR4. **a** Basophils were incubated with cell medium containing EDTA (4 mM) (*white histogram*) or CXCL12/SDF-1α (100 ng/ml) (*grey histogram*), for 30 min at 37 °C. At the end of incubation, basophils were washed twice, resuspended in Ca^2+^-containing buffer, and rechallenged with the chemotactic stimuli fMLF (500 ng/ml), CXCL12/SDF-1α (100 ng/mL), or r-Nef protein (100 ng/ml). *p < 0.05 as compared to control. **b** Basophils were incubated with cell medium containing EDTA (4 mM) (white histogram), fMLF (500 ng/ml) (light grey histogram), CXCL12/SDF-1α (100 ng/ml) (black histogram) or r-Nef protein (100 ng/ml) (*grey histogram*), for 30 min at 37 °C. At the end of incubation, cells were washed twice, resuspended in Ca^2+^-containing buffer, and challenged with the chemotactic stimuli CXCL12/SDF-1α (100 ng/ml) or r-Nef (100 ng/ml). Values are the mean ± SEM of three distinct experiments. *p < 0.05 as compared to basophils preincubated in the absence of chemotactic stimuli

The relationship between CXCR4 receptors and Nef protein was further examined using CXCL12/SDF-1α to induce desensitization of CXCR4-mediated functions. In a first series of experiments, purified basophils (>98%) were incubated with buffer containing EDTA (4 mM), alone or in in the presence of CXCL12/SDF-1α (100 ng/ml) for 30 min at 37 °C. At the end of incubation, basophils were washed twice, resuspended in Ca^2+^-containing buffer, and rechallenged with the chemotactic stimuli (fMLF 500 ng/ml, CXCL12/SDF-α 100 ng/ml or r-Nef 100 ng/ml). Figure 4a shows that the response to CXCL12/SDF-1α or r-Nef was significantly reduced by the preincubation of cells with CXCL12/SDF-1α. By contrast, CXCL12/SDF-1α desensitization didn’t affect fMLF-dependent chemotaxis.

In a second series of experiments, purified basophils (>98%) were incubated with a buffer containing EDTA (4 mM) in the presence or absence of CXCL12/SDF-1α (100 ng/ml) or r-Nef (100 ng/ml) for 30 min at 37 °C. At the end of the incubation, basophils were washed twice, resuspended in a Ca^2+^-containing buffer, and rechallenged with the chemotactic stimuli (fMLF 500 ng/ml, CXCL12/SDF-1α 100 ng/ml or r-Nef 100 ng/ml). Figure 4b shows that the response to CXCL12/SDF-1α was significantly reduced by the preincubation with homologous or heterologous stimuli. Similarly, preincubation with r-Nef significantly reduced the chemotactic activity of both CXCL12/SDF-1α and r-Nef, indicating that the two stimuli were using the same receptor. Again, the chemotactic response to fMLF was unaffected by the desensitization with CXCL12/SDF-1α or r-Nef.

### Effect of Nef on chemokine release from human basophils and mast cells

**Fig. 5:**
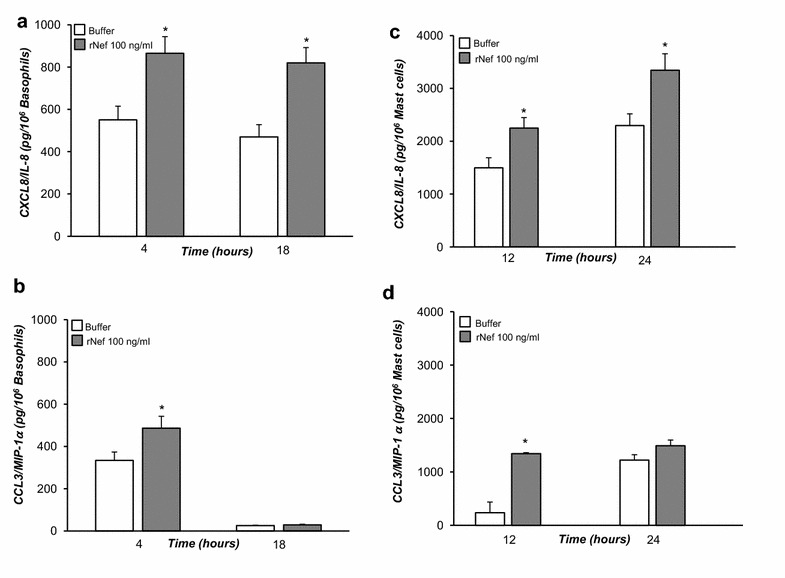
Effects of r-Nef on CXCL8/IL-8 and CCL3/MIP-1α release from human basophils and mast cells. **a** 10^6^ purified basophils/sample were incubated for 4 or 18 h without (*white histogram*) or with r-Nef (100 ng/ml) (*grey histogram*). Supernatants were collected at each time point. CXCL8/IL-8 was determined by ELISA. Values are the mean ± SEM of three distinct experiments. *p < 0.05 as compared to basophils preincubated in the absence of chemotactic stimuli. **b** 10^6^ purified basophils/sample were incubated for 4 or 18 h without (*white histogram*) or with r-Nef (100 ng/ml) (*grey histogram*). Supernatants were collected at each time point. CCL3/MIP-1α was determined by ELISA. Values are the mean ± SEM of three distinct experiments. *p < 0.05 as compared to basophils preincubated in the absence of chemotactic stimuli. **c** 10^6^ HLMC cells/sample were incubated for 12 or 24 h without (*white histogram*) or with r-Nef (100 ng/ml) (*grey histogram*). Supernatants were collected at each time point. CXCL8 was determined by ELISA. Values are the mean ± SEM of three distinct experiments. *p < 0.05 as compared to mast cells preincubated in the absence of chemotactic stimuli. **d** 10^6^ HLMC cells/sample were incubated for 12 or 24 h without (*white histogram*) or with r-Nef (100 ng/ml) (*grey histogram*). Supernatants were collected at each time point. CCL3/MIP-1α was determined by ELISA. Values are the mean ± SEM of three distinct experiments. *p < 0.05 as compared to mast cells preincubated in the absence of chemotactic stimuli

r-Nef upregulates mRNA for MIP-1α/MIP-1α and several cytokines in human monocytes/macrophages [37]. We tested whether r-Nef could induce chemochine release by human basophils, which are known to release CXCL8/IL-8 and CCL3/MIP-1α upon immunological activation [38]. We therefore evaluated, at different time-points, the release of CXCL8/IL-8 and CCL3/MIP-1α from basophils triggered with r-Nef. The results of three independent experiments showed a significant release of CXCL8/IL-8 after 4 h till 18 h of incubation (Fig. 5a), and CCL3/MIP-1α, after 4 h (Fig. 5b). Since the chemotaxis assay was performed after 1 h of incubation, it is likely that the chemotactic effect of Nef was not mediated by the release of chemokines from basophils. In addition we also evaluated the effects of increasing concentrations of Nef and CXCL12/SDF-1α on cytokine (IL-4 and IL-13) release from basophils purified from healthy donors. In five experiments, both r-Nef (10 and 100 ng/ml) and CXCL12/SDF-1α (10 and 100 ng/ml) did not cause cytokine release from these cells (data not shown). We finally evaluated the kinetics of chemokine release induced by r-Nef from HLMC cells. Similarly to basophils, r-Nef induced a significant release of CXCL8/IL-8 at 12 and 24 h (Fig. 5c) and of CCL3/MIP-1α at 12 h (Fig. 5d).

## Discussion

This study demonstrated that HIV-1 Nef protein is a chemoattractant for human basophils and mast cells (Fig. 2). The chemotactic activity of Nef protein was mediated by the interaction with the CXCR4 receptor present on a remarkable proportion of these cells (Figs. 1, 3). In addition, we found that Nef induced the production of chemokines (CXCL8/IL-8 and CCL3/MIP-1α) from basophils and mast cells (Fig. 5). This is the first demonstration that Nef protein is an HIV-1-encoded chemokine-homolog functionally active on human FcεRI^+^ cells through the interaction with the CXCR4 receptor.

It is well known that CXCR4 is a co-receptor for several strains of HIV-1 [39]. Here we demonstrated that soluble r-Nef specifically interacts with CXCR4 on human basophils and mast cells. Indeed, a monoclonal antibody anti-CXCR4 completely blocked the chemoattractant effect of Nef protein (Fig. 3). The specificity of this interaction was confirmed by the observation that preincubation of cells with anti-CCR3 antibody did not modify the chemotactic response of both r-Nef and CXCL12/SDF-1α (Fig. 3). Finally, the cross-desensitization of basophil chemotaxis with Nef provided the evidence that Nef interacts with the CXCR4 receptor on human FcεRI^+^ cells (Fig. 4).

These findings are relevant at different levels. Firstly, they suggest that during HIV-1 infection, Nef can influence the directional migration of human basophils and mast cells, thus contributing to the recruitment of these cells at sites of HIV-1 infection. Secondly, the chemotactic activity of Nef on human FcεRI^+^ cells might contribute to increase the local density of mast cells and basophils available for HIV-1 interaction through the virus-bound or shed gp120. In fact, we have previously demonstrated that gp120 from different clades interacts with the IgE V_H_3^+^ present on human FcεRI^+^ [26]. The superantigenic interaction between gp120 and IgE leads to the rapid synthesis and release of IL-4 and IL-13 from human FcεRI^+^ cells [18]. This interaction might represent an initial source of cytokines, thereby favoring a shift from a Th0 toward a Th2 phenotype. The latter observation is relevant because HIV-1 is known to replicate preferentially in Th2 cells [40]. Finally, mast cells and basophils rectruited at the site of HIV infection can directly contribute to the spread of the infection by acting as virus reservoir and mediating trans-infection of CD4^+^ T cells, as recently demonstrated [28, 30].

The clinical relevance of our findings is confirmed by the observation that Nef was present in the serum of HIV-1-infected patients at concentrations as high as 10 ng/ml [41]. In tissues where viral replication occurs (e.g. the lymph nodes), local levels of Nef could exceed those found in serum. Because the early phases of infection are associated with high levels of viremia [1], and this, in turn, may be associated with high levels of Nef, chemokine-like activity of Nef on FcεRI^+^ cells might be of clinical relevance in patients with HIV-1 infection.

Intriguingly, many viruses exploit the strategy of using homologs of cellular cytokines and chemokines to shield virus-infected cells from immune defenses and enhance virus survival in the host [42, 43]. The existence of these virus-encoded homologs of cellular proteins is indirect evidence of their relevant role in orchestrating the host immune response to invading pathogens [43]. Many large DNA viruses, including CMV and HHV-8, as well as the poxvirus *Molluscum contagiosum*, encode several α-chemokine homologs (virokines) acting on CCR3 or CCR8 receptors [44–46]. This novel observation may have several implications for a better understanding of the pathogenesis of HIV-1 infection.

In conclusion, we provided the first evidence that Nef protein is an HIV-1-encoded chemokine-homolog able to activate human FcεRI^+^ cells, by interacting with the CXCR4 receptor on these cells. Because HIV-1 enters the body predominantly through mucosal surfaces and because early phases of infection are associated with high levels of viremia, both mast cells, in tissues, and basophils, in circulation, can be exposed to high local levels of Nef protein, which in turns induce their recruitement and activation in sites of infection. Overall, our results suggests a novel mechanism through which FcεRI^+^ cells can contribute to the dysregulation of the immune system in HIV-1 infection.

## Abbreviations

CXCR4: C-X-C motif chemokine receptor 4
CCR5: C-C motif chemokine receptor 5
CCR3: C-C motif chemokine receptor 3
CXCL12/SDF-1α: C-X-C motif chemokine 12/stromal cell-derived factor 1-alpha
TNF: tumor necrosis factor
CXCL8/IL-8: C-X-C motif chemokine ligand 8/interleukine 8
CCL3/MIP-1α: C-C motif chemokine ligand 3/macrophage inflammatory protein 1-alpha
HSA: human serum albumin
anti-FcεRI: mouse monoclonal IgG anti-α chain of high affinity receptor for IgE
anti-IgE: rabbit IgG anti-Fc fragment of human IgE
FcεRI: high affinity receptor for IgE
HLMC: human lung mast cells
P: 25 mM PIPES (pH 7.4), 110 mM NaCl, and 5 mM KCl
FPRs: formyl-peptide receptors
fMLF: formyl-methionyl-leucyl phenylalanine
r-Nef: recombinant-Nef
